# Not All Free Air Needs a Scalpel: A Case of Spontaneous Pneumoperitoneum in a Patient With Asthma

**DOI:** 10.7759/cureus.84398

**Published:** 2025-05-19

**Authors:** Anas Dabsha, Michelle Feltes Escurra, Ameer Aboud, Sharique Nazir

**Affiliations:** 1 General Surgery, Harlem Hospital Center, New York, USA; 2 Minimally Invasive Surgery/General Surgery, New York City (NYC) Health + Hospitals/Harlem, New York, USA

**Keywords:** chronic obstructive pulmonary disease (copd), conservative and surgical treatment, free air under the diaphragm, idiopathic spontaneous pneumoperitoneum, surgical acute abdomen

## Abstract

Pneumoperitoneum typically suggests gastrointestinal perforation requiring urgent surgery. However, in rare cases, it occurs without intra-abdominal pathology. We present a case of a 55-year-old male with asthma who developed spontaneous pneumoperitoneum. He presented with respiratory symptoms and mild abdominal discomfort; imaging revealed free intraperitoneal air. With a benign abdominal exam and clinical stability, he was managed conservatively. This case emphasizes distinguishing surgical from non-surgical pneumoperitoneum to avoid unnecessary operations, particularly in patients with pulmonary conditions. Clinicians should maintain awareness of non-surgical and intrathoracic causes to ensure accurate diagnosis and appropriate care.

## Introduction

Pneumoperitoneum (free air in the peritoneal cavity) is usually a marker of gastrointestinal perforation, with about 90% of cases requiring surgery. However, approximately 10% of the results are from non-surgical causes, including thoracic, gynecologic, or iatrogenic origins, or may occur spontaneously [1]. Idiopathic pneumoperitoneum, a diagnosis of exclusion, is rare and identified when no clear source is found despite evaluation [2,3]. Early recognition is crucial, as such cases may be managed nonoperatively, sparing patients unnecessary surgery [4]. While exploratory laparotomy can be lifesaving, it carries significant morbidity and mortality. Avoiding non-therapeutic laparotomy requires careful diagnosis and clinical judgment [5]. We present a case of a patient with benign abdominal findings and radiologic pneumoperitoneum successfully managed without surgery.

## Case presentation

A 55-year-old male with asthma and hypertension and no prior abdominal surgeries presented to the emergency department with pleuritic chest pain, a productive cough with yellow sputum for three days, and mild abdominal discomfort for one week. He denied nausea, vomiting, diarrhea, or constipation.

**Figure 1:**
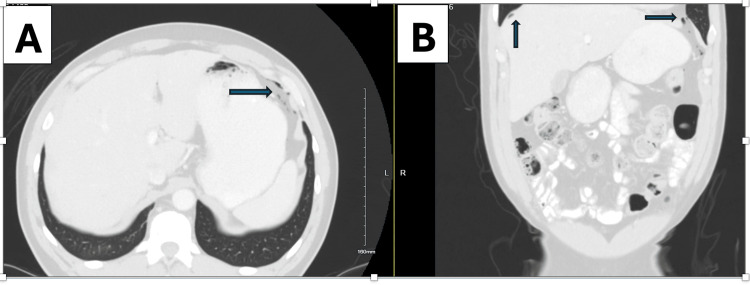
Axial view (A) and (B) coronal view CT scan images showing free intraperitoneal air in axial and sagittal views, arrows pointing at the free air CT: computed tomography

On examination, his abdomen was soft, non-tender, and non-distended. He had undergone a normal colonoscopy one year earlier. A chest X-ray revealed a subdiaphragmatic lucency consistent with pneumoperitoneum. Abdominal and pelvic CT with IV contrast showed a small amount of free intraperitoneal air and colonic diverticulosis without diverticulitis or definitive perforation (Figure 1). He tested positive for respiratory syncytial virus, and WBC was normal (5.13). Given his stable vitals and benign abdominal exam, a nonsurgical cause was favored.

He was admitted for observation, kept NPO, and treated supportively with IV piperacillin-tazobactam 4.5 g every six hours. Over 24 hours, he remained stable with unchanged abdominal exams. His diet was advanced gradually without issue. He was discharged in good condition and scheduled for outpatient follow-up. Informed consent was obtained from the patient for case presentation and publication purposes.

## Discussion

Exploratory laparotomy is a significant decision in surgical care, requiring careful discrimination between patients who need surgery and those who do not. Pneumoperitoneum is commonly due to gastrointestinal perforation, but in about 10% of cases, it arises from non-surgical causes [6,7].

Non-surgical pneumoperitoneum has been reported from intrathoracic, gastrointestinal, gynecologic, and iatrogenic sources [8]. For example, pneumatosis cystoides intestinalis, characterized by gas-filled cysts in the bowel wall, can rupture spontaneously without true perforation [9,10].

Intrathoracic causes occur when air dissects from ruptured alveoli (e.g., due to coughing or asthma) into the mediastinum (Macklin effect) and then into the peritoneal cavity via diaphragmatic openings such as the esophageal hiatus or the foramen of Morgagni [11]. Diaphragmatic microdefects or elevated intrathoracic pressure may facilitate this process [12].

Gynecologic causes are exclusive to females due to anatomical communication with the peritoneum. Reported sources include pelvic inflammatory disease, douching, sexual activity (particularly post-hysterectomy), and physical exertion [13,14]. Rare causes such as scuba diving and hot tub use have also been noted [15,16]. Radiologic mimics, such as the Chilaiditi sign, interposition of bowel between the liver and diaphragm, must be ruled out [17].

Our patient, with asthma and no abdominal pathology, presented with respiratory symptoms and pneumoperitoneum. His benign abdominal exam supported a conservative approach. This case reinforces the association between chronic pulmonary disease and spontaneous pneumoperitoneum and supports a nonoperative pathway when clinical signs are reassuring.

## Conclusions

Spontaneous pneumoperitoneum without peritonitis can occur in patients with chronic pulmonary disease. Awareness of this entity allows clinicians to avoid unnecessary surgery when clinical findings are benign. Accurate diagnosis and sound clinical judgment are essential for optimal management. This case underscores the potential association between chronic pulmonary disease and pneumoperitoneum of thoracic origin. Further studies are warranted to elucidate the pathophysiology better, improve diagnostic accuracy, and develop risk-stratification tools to guide appropriate management in these rare presentations.
